# Three Years with the Army of the Potomac—A Personal Military History

**Published:** 1912-05

**Authors:** William Warren Potter

**Affiliations:** Buffalo, N. Y., Brevet Lieutenant Colonel, U. S. Volunteers; Surgeon in Charge of First Division Field Hospital Second Army Corps; Surgeon 57th Regiment New York Volunteers; Assistant Surgeon 49th Regiment New York Volunteers; Recorder Second Division Hospital Sixth Army Corps, etc., etc.


### Three Years with the Army of the Potomac—A Personal Military History

BY WILLIAM WARREN POTTER, M.D., BUFFALO, N. Y., BREVET LIEUTENANT COLONEL, U. S. VOLUNTEERS; SURGEON IN CHARGE OF FIRST DIVISION FIELD HOSPITAL SECOND ARMY CORPS; SURGEON 57TH REGIMENT NEW YORK VOLUNTEERS; ASSISTANT SURGEON 49TH REGIMENT NEW YORK VOLUNTEERS; RECORDER SECOND DIVISION HOSPITAL SIXTH ARMY CORPS, ETC., ETC.

November 1, 1863. This has been one of the finest days imaginable, just the day for marching, and we are expecting orders.

November 3. It was certainly anticipated that we should move before this, and I hear that a movement is on the tapis which is likely to occur very soon. The weather could not be more delightful, Indian Summer has set in, the trees are losing their foliage amidst their everchanging hues, and everything looks autumn-like, much as it does North a month earlier. I expect to return to my regiment in a few days if we do not move, as I have nothing to do here.

November 5. The weather continues pleasant, but orders do not come for us to move yet. Yesterday, however, the sick were all sent to Washington—a pretty sure indication that we shall march soon.

Tomorrow night (Friday, November 6, we intend giving a party at this camp, providing, of course, we do not move before. Our headquarter tents are trimmed with evergreens, a band of music is engaged, and all preparations are completed for a jolly time. Last night (the 4th) the Second Delaware Volunteers gave a party, where a large number of the officers met and had a very enjoyable time.

November 9. We broke camp at Warrentown, or near there, day before yesterday, the 7th, early in the morning, and marched all day in a cloud of dust. Yesterday morning early we crossed the Rappahannock at Kelley's Ford, without much opposition.

The 6th Corps crossed at Rappahannock Station, taking 1,100 prisoners, and four cannons, in a redoubt at the R. R. Bridge. We are not anticipating the enemy will make much of a stand this side of the Rapidan. Our party came off on Friday night as planned, though it broke up at midnight on account of the orders to move next morning early. We had about fifty guests, good music, a collation, Milk Punch, et ceteras; and everybody was happy, or seemed to be, at least.

November 12. This is Thursday, and for two or three days past it has been cold with a little snow; but this morning it is warmer and consequently more pleasant. We have been constantly changing position for the past three or four days, but nothing has been accomplished beyond getting the several corps well into line.

November 14. Saturday. There is a feeling afloat that we shall move on Monday next, but it is difficult to say on what it is based.

November 16. Monday. A terrific rain storm passed over us last night, but this morning it is pleasant. Firing has been heard at intervals during the day, along our front and that of the right wing, owing to an attack of the rebel cavalry, in which they are reported to have driven ours back upon the Sixth Corps. We are ordered to hold ourselves in readiness to move at a moment's notice, but I don't anticipate a general engagement until this army moves forward again, which is likely to be sometime during the week. I shall not return to my regiment as I thought probable a few days ago, as there is something to do here as long as we are actively campaigning.

November 17. We are near Brandy Station. The weather is very fine now, and we shall probably move soon. In regard to the cavalry fight of Sunday last (the 15th), it is said by some that we got the worst of the bargain, and lost 500 men; I rather think we were beaten, but cannot learn the particulars.

November 19. We are still here, hourly expecting orders. Some English officers were entertained yesterday with a review and drill of the Second Corps, and expressed themselves as much pleased with the American soldiery.

November 21. A drizzling rain has kept up since daylight, and, as a matter of course, the mud is pretty deep tonight.

November 23. We are ordered to move tomorrow at daylight.

November 25. We did not move yesterday as ordered. Our tents were struck, wagons packed, and the head of the ambulance train pulled out for the start—all in the midst of a heavy rain—when the orders came countermanding the movement.

Every man cheered lustily, and the woods for miles rang with the echoes of applause. The mud is knee deep, or thereabouts, which is the explanation of the change of orders; but I presume the movement is only temporarily in abeyance. The rain is still pattering on my tent, but as soon as the roads are sufficiently dry we may expect to be off. Tomorrow will be Thanksgiving Day, and our dinner will consist of pork and beans, bread without butter, and no special viand.

### Mine Run.

On Thanksgiving Day, Thursday, November 26, 1863, we broke camp at daylight and marched to Germanna Ford on the Rapidan, a distance of seven miles, which we had reached before noon. The enemy made no resistance to our crossing there, but, owing to the steep banks on either side of the river, it was late at night before the entire corps, including the artillery, had crossed, some of the latter not getting over until the next morning. We camped at nine o'clock at night about two miles beyond the Rapidan, on the road leading from Culpepper C. H. to Fredericksburg, and about five miles from Robertson's Tavern. By seven o'clock on the morning of the 27th, the corps was well under way, the Second Division leading, the Third Division next, and the First Division bringing up the rear. By ten o'clock the skirmishers had encountered the enemy, and a rattling fire of musketry was kept up for two hours, the rebels falling back to Robertson's Tavern on the Orange and Fredericksburg Pike. Skirmishing was kept up until nightfall, with occasional discharges of artillery, and the casualties of the Corps had reached, probably, fifty men. During the night of the 27th, Friday, the enemy fell back to Mine Run, where he was strongly fortified.

We followed at early daylight, and the 28th was spent very much as the day previous, viz.: in skirmishing, and feeling the position. On the morning of the 29th the Second Corps, with one Division of the Sixth, all under command of General Warren, moved by a circuitous route around to the enemy's right flank, with the design of turning his position. We arrived there about five P. M., too late to accomplish much that night, and the next morning (30th) it was discovered that their fortifications there were even stronger than those we had left in the center. We spent two days in skirmishing and feeling, and at nine o'clock P. M., December 1, turned our backs upon the "Rebs" and their fortified position, and commenced retracing our steps toward our old camps. We marched all night in the clear, cold air, suffering therefrom intensely, and by daylight, Tuesday, December 2, the whole army was safely on the north side of the Rapidan again.

This is a brief sketch of what we actually did, and I shall not attempt to say what we might have done. It is sufficient to remark that the Army feels entirely satisfied with General Meade's decision not to attack such formidable works as he found, no matter what the people of the North may say about it. We had no communication with Washington for the eight days we were away.

On Friday, December 4, I was sent to Washington with a R. R. train load of wounded from the Second Corps, reaching the city at midnight, remaining till the 8th.

Wednesday, December 9. I left Washington yesterday morning, but did not reach camp until this forenoon. I arrived at Brandy Station at a late hour last night, and was kindly offered quarters by Dr. J. Bernard Brinton, the Army Medical Purveyor. I enjoyed the brief visit to the capital, for it is a relief to get away from the Army even for a few days; especially after such a severe campaign as we recently had over the Rapidan. The weather was extremely cold. We could have no fires that were at all comfortable, and the Army suffered severely. I don't think we will make another aggressive campaign this winter, but we shall probably move to a more favorable spot for winter quarters, where timber and water can be had more plentifully; here there is very little of either.

December 11. The weather is very cold and we haven't even a comfortable place to write a letter, but by tomorrow night we expect to be comfortably domiciled in a log house we are building. The weather reminds me much of the season North; being about the same temperature. An order was issued last night restoring the ten days leave of absence to officers. One surgeon can be absent from each Brigade at a time, and this may give me an opportunity to visit home in January.

December 16. Rumors were rife yesterday that we should move today, but the day has nearly passed and no orders have yet been received indicative of a movement. Many officers are of the opinion we will stay here during the winter, but I have not yet settled into that belief.

December 18. It has rained almost uninterruptedly for thirty-six hours, and the roads, as a consequence, are simply horrible; in fact, one cannot step outside of quarters without going ankle-deep in the mud. I have not been outside of camp for five days, and don't attempt to go anywhere except on urgent business. This would be considered lazy in the North, but it is not so here, where one becomes so completely ennervated after a campaign of deprivation and suffering.

December 20. Today is a cold, bright winter day, which is an agreeable change after the rain of the past week.

December 23. Yesterday I visited the Sixth Corps and the 49th, where I saw Colonel Bidwell, Dr. Hall, Major Ellis, and other officers of my acquaintance. Major Ellis went away last night for ten days, leaving in his usual good spirits. Today is a pleasant, cold day, much like a December day in the North. It is my present purpose to apply for a leave about January 10, prox., which will fetch me home about the 15th or 18th.

December 26. Christmas Day passed off yesterday in a very quiet manner with us. We dined on roast turkey, and had porter for drink. Dr. B. Gesner, Medical Inspector of the Second Corps, was our only guest. He has been recently married, and proposes fetching his wife down to the Army next week. If I should have a hospital, as I now expect, perhaps I will not ask for leave, but have my wife come and visit me instead.

December 28. We are still at the same place, but I may leave the Ambulance Corps in a few days if I take charge of the Division Hospital again. It has been raining very hard for twenty-four hours.

December 29. Division Hospitals have been ordered re-established, and I have been appointed to the charge of the First Division Hospital. I have drawn ten new tents today, and shall commence putting them up tomorrow; in the course of another week everything will be in running order. Several ladies have already arrived in camp, and more are expected soon. Dr. Gesner goes north tomorrow to fetch his wife, and every train is bringing the women into camp. General Meade has issued an order permitting the wives, sisters or mothers of officers to visit the Army for twenty days, and the order is being complied with most heartily.

